# Naringenin Mitigates Dasatinib-Induced Kidney Damage by Modulating Antioxidant Defense, Inflammation, and Apoptosis Pathways

**DOI:** 10.7150/ijms.102088

**Published:** 2025-01-01

**Authors:** Khalid Alhazzani, Naif N. Alqarni, Khaldoon Aljerian, Mohammad Raish, Lobna Aljuffali, Samiyah Alshehri, Ahmed Z. Alanazi

**Affiliations:** 1 Department of Pharmacology and Toxicology, College of Pharmacy, King Saud University, Riyadh, Saudi Arabia.; 2 Department of Pathology, College of Medicine, King Saud University, Riyadh, Saudi Arabia.; 3 Department of Pharmaceutics, College of Pharmacy, King Saud University, Riyadh, Saudi Arabia.; 4 Department of Clinical Pharmacy, College of Pharmacy, King Saud University, Riyadh, Saudi Arabia.

**Keywords:** Naringenin, Dasatinib, Nephrotoxicity, Oxidative stress, Inflammation, Apoptosis

## Abstract

Nephrotoxicity remains a significant concern associated with tyrosine kinase inhibitors, such as dasatinib (DASA). Previous studies have shown that DASA can induce renal tubular cell death, contributing to its nephrotoxic effects. In contrast, naringenin (NGN) is known for its antioxidant and anti-inflammatory properties. This study aimed to explore the nephroprotective potential of NGN against acute kidney injury induced by DASA in a mouse model. Mice were pre-treated with different doses of NGN (50, 100 mg/kg) for one week, followed by a single dose of DASA (25 mg/kg) on the 8th day. Results demonstrated that DASA significantly increased serum levels of blood urea nitrogen, creatinine, uric acid, and lactate dehydrogenase, which were effectively attenuated by NGN pretreatment. Furthermore, kidney tissues exposed to DASA exhibited elevated malondialdehyde (MDA) levels, which were significantly reduced by NGN. NGN also restored depleted levels of antioxidants (glutathione (GSH) and catalase (CAT)) in kidney tissues following DASA treatment. Additionally, NGN mitigated the upregulation of pro-inflammatory cytokines (TNF-α, NF-κB, and IL-6) induced by DASA, indicating an anti-inflammatory effect. Notably, DASA treatment upregulated the gene expression of the pro-apoptotic gene BAX while downregulating the expression of BCL-2 and Caspase-3 in kidney tissues. These findings suggest that NGN exerts nephroprotective effects against DASA-induced nephrotoxicity through its antioxidant, anti-inflammatory, and anti-apoptotic properties. Further investigations are warranted to elucidate the underlying mechanisms involved.

## Introduction

Acute kidney injury induced by anti-cancer treatment is becoming increasingly prevalent^1^,^2^. Nephrotoxicity, in fact, has emerged as a significant factor that hampers the effectiveness of anticancer agents 3,4. This is primarily because the kidneys serve as the primary site for the excretion of these agents and their metabolites. It occurs when normal kidney functions, including filtration, detoxification, and excretion, are compromised due to damage inflicted on the kidneys' architecture 5. Such damage can range from reversible mild to severe permanent damage, which may result in systemic toxicity due to delays in drug excretion and metabolism. Therefore, treatment cessation or dose adjustment might be required if kidney function is significantly impaired^6^.

Despite improvements in cancer treatment, targeted therapy, including tyrosine kinase inhibitors, is still associated with nephrotoxicity^7^. Dasatinib (DASA) is an effective second-generation tyrosine kinase inhibitor for the treatment of leukemia by targeting the breakpoint cluster region-Abelson (BCR-ABL) kinase. Despite its therapeutic efficacy, DASA might cause serious renal adverse effects. A meta-analysis study showed that DASA is associated with a high risk of nephrotoxicity, specifically glomerular toxicity, with no increase in hypertension risk^8^. This suggests that DASA may directly cause kidney damage, leading to glomerular injury, rather than as a secondary effect of hypertension. Furthermore, DASA has been shown to disrupt the actin cytoskeleton architecture and morphology in cultured murine podocytes, suggesting glomerular dysfunction and nephrotoxicity^8^. In clinical settings, DASA has been associated with acute renal failure and proteinuria. The severity of DASA-induced proteinuria seems to be dependent on the dosage 9,10. Hence, there is a need to find a new effective treatment that mitigates DASA-induced renal damage.

Naringenin (NGN), a common dietary flavonoid found in a variety of fruits, has been studied for its pharmacological effects. Its clinical use is broad, owing to its antioxidant and anti-inflammatory properties 11. NGN has been shown to mitigate oxidative stress and inflammation, contributing to its protective effects against hepatic, neurological, and cardiovascular diseases 12. Studies have shown that NGN exhibits protective effects against neurodegenerative diseases by reducing oxidative stress and inflammation in the brain 13. It also protects against cardiovascular diseases by improving endothelial function, reducing oxidative stress, and inhibiting inflammation 12. Moreover, NGN has been shown to protect against liver and kidney damage induced by various toxins, primarily through its antioxidant and anti-inflammatory effects 11,14. NGN has been found to decrease the expression of several mediators of inflammation, such as cyclooxygenase-2, TNF-α, IL-β, and IL-6 15. Additionally, NGN has demonstrated protective effects in animal models, such as protecting C57BL/6J mice from the detrimental effects of obesity by significantly decreasing fat weight, liver weight, and lipid profile 16. Furthermore, NGN has been shown to reduce pro-inflammatory cytokines such as IL-6, TNF-α, and NF-κB, which are believed to have protective effects against azoxymethane/dextran sodium sulfate-induced colorectal cancer in male C57BL/6 mice 17.

However, the effects of NGN on DASA-induced nephrotoxicity have not been investigated. Therefore, this study aims to investigate the renal protective effect of NGN against DASA-induced nephrotoxicity by measuring serum levels of kidney function biomarkers. Additionally, we will explore the mechanisms by which NGN exerts its protective effects by assessing the impact of NGN ± DASA treatment on the biomarker levels of antioxidants, inflammation, and apoptosis in renal tissues.

## Materials and Methods

### Materials

DASA and NGN were obtained from MedChemExpress (Junction, NJ, USA), and Sigma-Aldrich (St. Louis, MO, USA), respectively. Gene primers were purchased from Integrated DNA Technologies (Leuven, Belgium). Serum diagnostic kits for creatinine, lactate dehydrogenase, uric acid, and blood urea nitrogen were purchased from Linear Chemicals (Barcelona, Spain). For the experimental procedures, DASA was freshly prepared by dissolving it in a 0.5% carboxymethyl cellulose solution.

### Animal and Housing Conditions

Male Swiss albino mice (aged 6-8 weeks, weighing 25-30 g) were deployed to investigate the impact of NGN and DASA on renal function and structure. These mice were obtained from the Experimental Animal Care Center at the College of Pharmacy, King Saud University (KSU), Riyadh, Saudi Arabia. They were housed under standard animal housing conditions, with unrestricted access to water and a standard pellet diet.

### Experimental Design

To achieve the study objectives, mice were randomly assigned into 4 groups, each consisting of six mice. The groups were as follows:

1. Control group, which received 0.5% carboxymethyl cellulose as a vehicle.

2. DASA group, which received 25 mg/kg via oral gavage.

3. NGN50 + DASA; which received 50 mg/kg of NGN via oral gavage for seven consecutive days, followed by a single dose of 25 mg/kg DASA on the eighth day.

4. NGN100 + DASA, which received 100 mg/kg of NGN via oral gavage for seven consecutive days, followed by a single dose of 25 mg/kg DASA on the eighth day.

Post-DASA administration, the mice were fasted overnight and subsequently anesthetized with a ketamine/xylazine mixture on the ninth day. Kidney tissue and serum were then collected for further biochemical and histological analyses.

### Biochemical Analysis

Blood samples of the experimental groups were collected from orbital sinus. These samples were then centrifuged at a speed of 800 g for a duration of 10 minutes to segregate the blood components and extract the serum. The serum was then analyzed for creatinine, uric acid, lactate dehydrogenase, and blood urea nitrogen levels, which serve as key biomarkers of kidney function. These measurements were conducted using a colorimetric method following the manufacturer's protocol.

### ELISA

After euthanizing the mice, kidney samples were promptly immersed in liquid nitrogen for one minute and then stored in a -80°C freezer. To extract kidney proteins, the kidney tissue was homogenized in a cold PBS buffer, which facilitated the disintegration of tissue architecture and cell lysis. The total protein concentrations in the kidney samples were determined using bicinchoninic acid (BCA) method with bovine serum albumin as a standard. The levels of TNF-α, IL-6, and MDA in the kidney were assessed using ELISA techniques (Thermo Scientific, Rockford, IL, USA). Enzymatic activities of CAT and GSH in the post-mitochondrial supernatants of kidney samples were measured using ELISA kits following the manufacturer's protocol.

### Histological Examination

The histological evaluation was carried out to investigate any alterations in kidney morphology and architecture post-treatment. In brief, the harvested kidney tissues were promptly fixed in a 10% formalin solution to preserve the tissue for subsequent histopathological examinations. Post-fixation, the kidney tissues underwent a dehydration process and were then embedded in paraffin wax. This allowed for the tissues to be sectioned into 4 µm slices using an automated microtome (Leica RM 2125, Leica Microsystems, Nussloch, Germany). These thin tissue sections were then mounted onto glass slides. All the slides were stained with hematoxylin and eosin (H&E) for enhanced visibility of the tissue structures. The stained slides were then examined under a Nikon Eclipse E600 light microscope equipped with a high-resolution digital camera. An experienced pathologist conducted the examination and analysis of the slides.

### Semiquantitative Assessment of Kidney Damage

To estimate the degree of kidney damage in histopathological samples stained with H&E, we employed a semiquantitative scoring system adapted from the study by Elsayed, H.R.H. *et al.* 18, with modifications suited to the characteristics of our samples. The assessment was performed on five randomly selected microscopic fields per sample, focusing on the following characteristics: significant infiltration of neutrophils, lymphocytes and plasma cells, mesangial hypercellularity, glomerular space enlargement, and dilated tubules. Each characteristic was scored based on the percentage of involvement within the observed fields, using the following grading scale (**Table 1**).

### RNA Extraction and Real-Time PCR Analysis

Total RNA from kidney tissue was isolated using the TRIzol method. The quantity and quality of the extracted RNA was examined by analyzing the ratio of OD 260/280 using NanoDrop 8000 (Thermo Fisher Scientific, Waltham, MA, USA). Then, RNA was reverse transcribed into cDNA using MedChemExpress kits (New Jersey, USA). The mRNA expression changes of BAX, BCL-2, and Caspase-3 genes in response to DASA, with or without NGN treatment, were quantified using the Real-Time PCR System and SYBR Green Master Mix (MedChemExpress, New Jersey, USA). The data were presented as a fold change in gene expression, normalized to the endogenous reference gene (GAPDH), using the 2 delta-delta threshold cycle method (2^- ΔΔCT^ method).

### Statistical Analysis

Statistical analysis was performed using GraphPad Prism software. Data are presented as the mean with error bars representing the standard deviation. We assessed the normality of the data using the Shapiro-Wilk test. All datasets, except for the data presented in Table 2, met the assumption of normality; therefore, one-way ANOVA was applied to these datasets, followed by Tukey's *post hoc* test for multiple comparisons. For the data in Table 2, which did not pass the normality test, we utilized the Kruskal-Wallis test, presenting results as median and interquartile range. Statistical significance was determined at a p-value of less than 0.05 (p<0.05), with (*) indicating p<0.05, (**) indicating p<0.01, and (***) indicating p<0.001.

## Results

### Impact of NGN on Serum Biomarkers of Kidney Function

**Figure 1:**
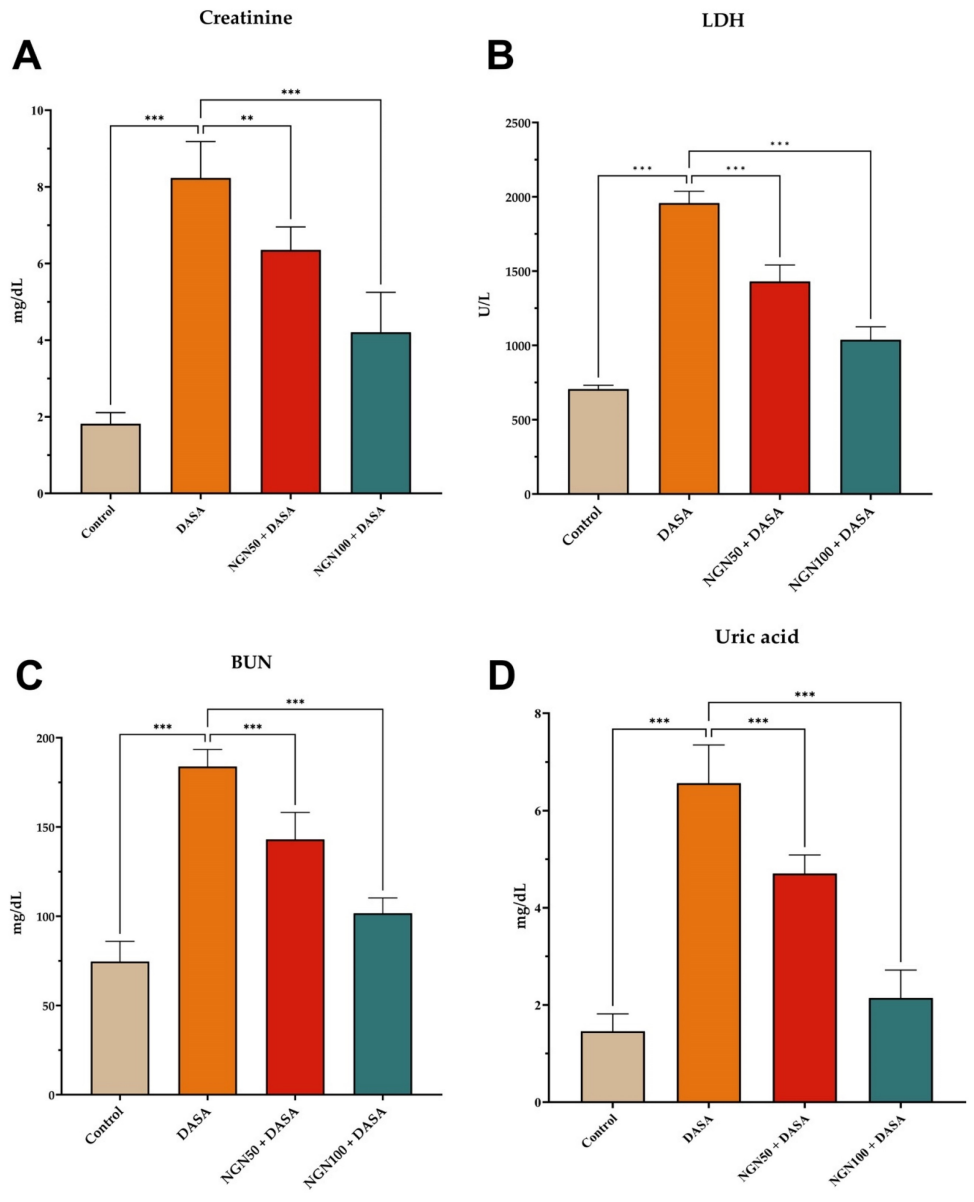
** Effects of NGN on serum biomarkers of kidney function in DASA-induced renal damage.** The figure presents the levels of **(A)** creatinine, **(B)** lactate dehydrogenase (LDH), **(C)** blood urea nitrogen (BUN), and **(D)** uric acid in different experimental groups. The DASA-treated group demonstrated a significant elevation in serum levels of creatinine, BUN, LDH, and uric acid compared to the control, indicating renal damage. However, when NGN was administered in combination with DASA, there was a significant dose-dependent reduction in the levels of these markers.

In this study, the serum biomarkers of creatinine, lactate dehydrogenase (LDH), blood urea nitrogen (BUN), and uric acid were assessed to evaluate the impact of NGN on DASA-induced renal damage **(Figure 1)**. The DASA-treated mice showed a significant increase in serum levels of creatinine, LDH, BUN, and uric acid compared to the control group, indicating renal damage. However, when NGN was administered in combination with DASA, there was a notable reduction in the serum levels of these markers. The extent of reduction was found to be dose-dependent on NGN. Furthermore, LDH, an enzyme present in various tissues including the kidneys, was measured as an indicator of tissue damage **(Figure 1B)**. The results showed that DASA treatment led to elevated levels of LDH in the blood, suggesting kidney damage.

### The Impact of NGN on Structure and Morphology of Kidney Tissues

**Figure 2:**
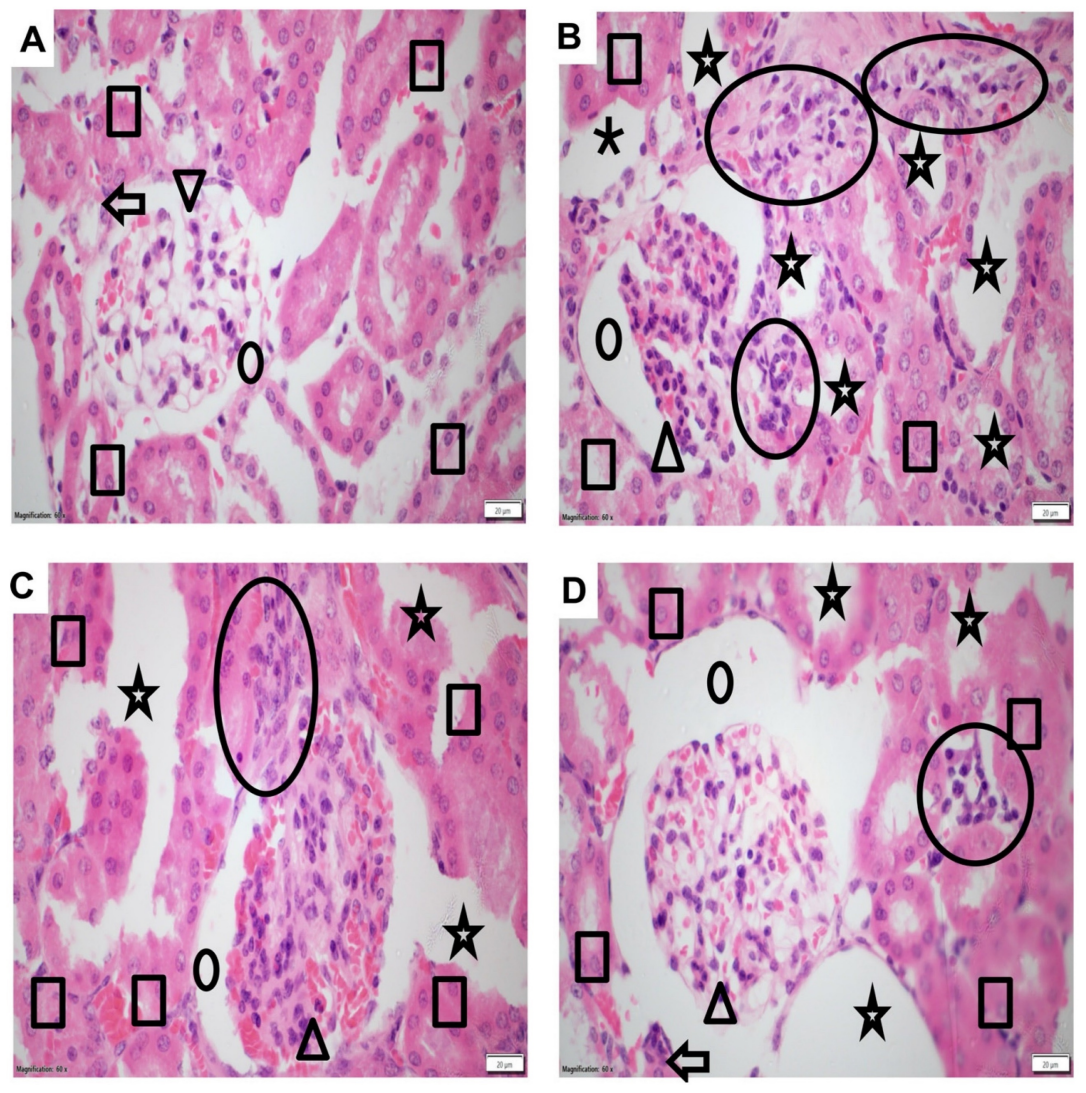
** Histopathological changes in kidney tissues of experimental mice. (A)** The control group exhibits normal kidney tissue morphology and architecture, showing no signs of inflammation. **(B)** The DASA group displays acute inflammation, characterized by significant infiltration of neutrophils, lymphocytes, and plasma cells, indicating kidney damage. **(C)** The administration of NGN (50 mg/kg) along with DASA resulted in moderate mesangial hypercellularity, increased mesangial matrix (arrowhead), moderate glomerular space enlargement (dot), moderate inflammation (circle), and moderate dilated tubules. **(D)** The administration of NGN at a dose of 100 mg/kg along with DASA further enhances the protective effect, resulting in improved recovery and alleviation of kidney tissues from the damaging effects of DASA. In H&E images, the square indicates tubules, the dot represents glomerular space, the arrowhead points to the glomerulus, the arrow marks the arteriole, and the circle highlights areas of inflammation.

To investigate the structural and morphological changes in kidney tissues, samples from different treatment groups were subjected to histopathological analysis using hematoxylin and eosin staining. The control group displayed intact tissue morphology and architecture, indicating the absence of inflammation or any abnormalities **(Figure 2A)**. Kidney samples from mice treated with DASA exhibited pronounced inflammation, evident by significant enlargement of glomerular space and dilated tubules, in comparison to the control group **(Figure 2B)**. However, the effectiveness of low and high doses of NGN in mitigating DASA-induced kidney damage was illustrated in **Figures 2C-D.** The histopathological analysis indicated that the co-administration of NGN with DASA led to enhanced recovery and alleviation of kidney tissues from the detrimental effects caused by DASA treatment.

The histopathological scores among the groups showed a significant difference, with higher scores in the DASA group (median score = 4) when compared to the control group (median score = 0). There was a significant reduction in the NGN 50 + DASA group (median score = 2) and the NGN 100 + DASA group (median score = 1) when compared to the DASA group (**Table 2**).

### Effects of NGN on Antioxidant Levels in Kidney Tissues

**Figure 3:**
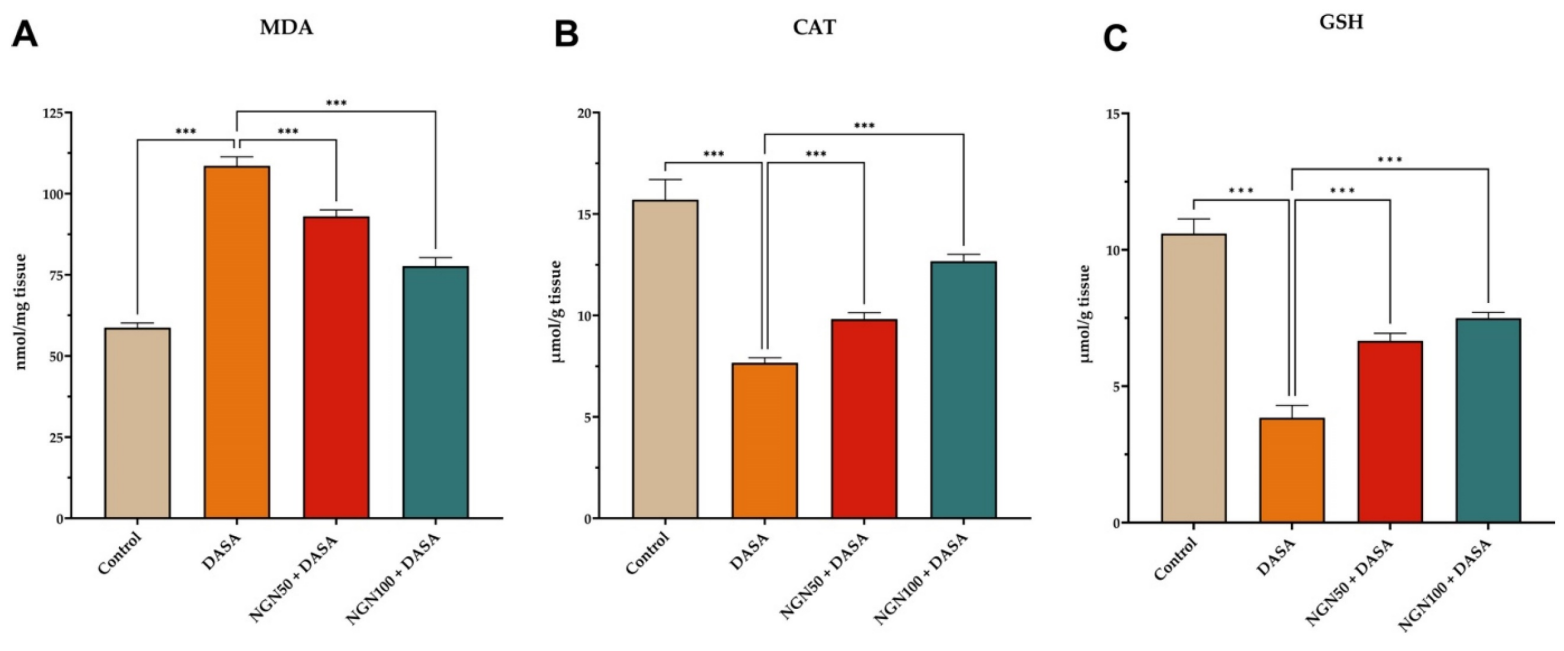
**Effects of NGN on antioxidant levels in kidney tissues.** The figure illustrates the quantification of **(A)** Malondialdehyde (MDA), **(B)** Catalase (CAT), and **(C)** Reduced Glutathione (GSH) levels. DASA administration induces oxidative stress in the kidneys, as indicated by elevated levels of MDA and reduced levels of CAT and GSH. This suggests that DASA treatment leads to oxidative stress in the kidneys. However, pretreatment with NGN at doses of 50 and 100 mg/kg in combination with DASA effectively reduces MDA levels, demonstrating its potential to mitigate oxidative stress and lipid peroxidation.

In order to investigate the impact on antioxidant levels, the study measured the levels of malondialdehyde (MDA), catalase (CAT), and reduced glutathione (GSH) in kidney tissue from different experimental groups. The results revealed that DASA administration led to increased oxidative stress, as evidenced by elevated MDA levels, along with reduced CAT and GSH levels in kidney tissues **(Figure 3)**. These findings indicate that DASA treatment induces oxidative damage in the kidneys. Furthermore, we investigated whether NGN could mitigate the oxidative stress induced by DASA treatment. As depicted in **Figure 3**, NGN significantly reduced the levels of MDA compared to mice treated with DASA alone. MDA serves as a commonly used biomarker for oxidative stress and lipid peroxidation. Moreover, DASA treatment resulted in a reduction of GSH and CAT levels. However, pretreatment with NGN (50 and 100 mg/kg) in combination with DASA showed a significant increase in GSH and CAT levels compared to DASA treatment alone. This suggests that NGN has the potential to counteract the decline in GSH and CAT levels induced by DASA, thereby preserving antioxidant capacity in the kidneys.

### Impact of NGN on Pro-inflammatory Cytokine Expression

**Figure 4:**
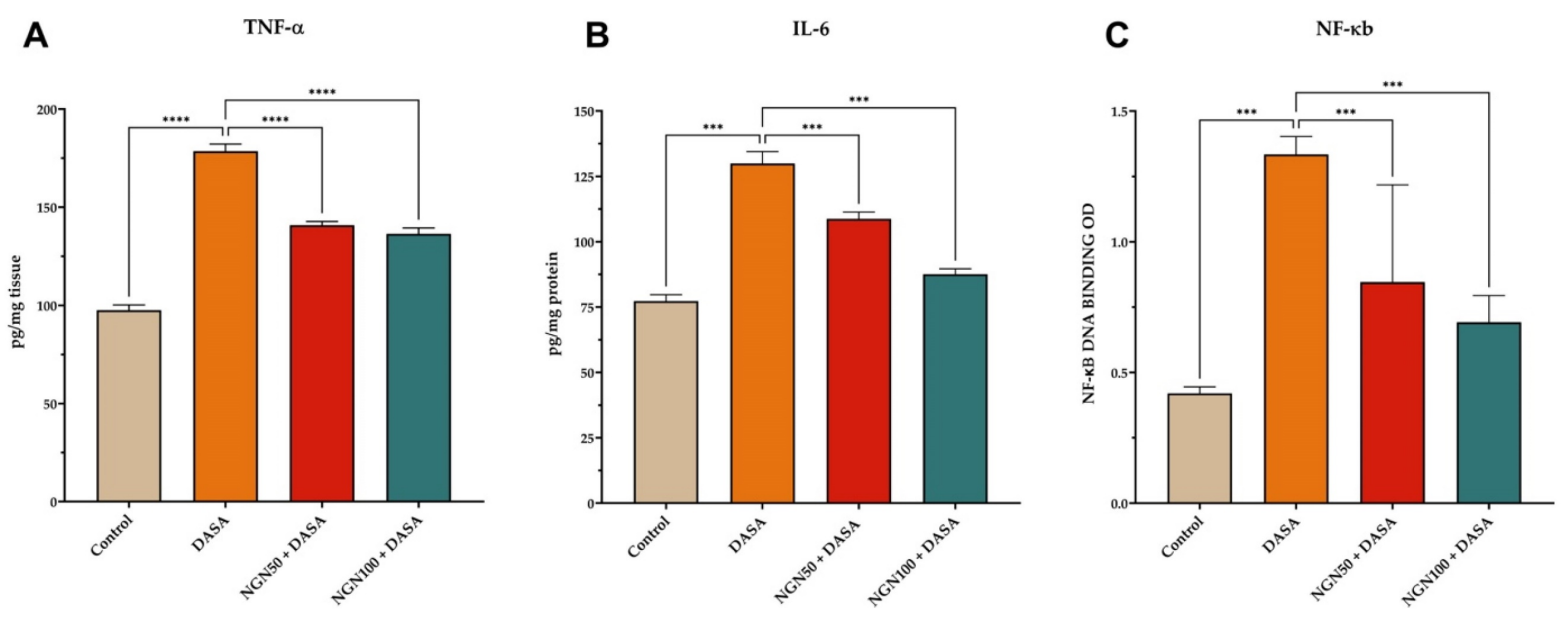
** Impact of NGN and DASA on pro-inflammatory cytokine expression in kidney tissues.** The figure illustrates the alterations in **(A)** TNF-α, **(B)** IL-6, and **(C)** NF-κB levels in kidney tissue samples across various treatment groups. DASA treatment leads to a significant increase in the levels of pro-inflammatory cytokines compared to the control group. This indicates the induction of an inflammatory response in the kidneys by DASA. However, pretreatment with NGN demonstrates a significant reduction in the levels of proinflammatory cytokines, including TNF-α, IL-6, and NK-κB, when compared to DASA treatment alone.

Kidney inflammation can lead to the destruction of kidney structure, which eventually compromises kidney function. To investigate the changes in kidney function, samples of kidney tissue obtained from various treated groups were subjected to ELISA analysis to detect changes in protein expression. DASA treatment resulted in a significant increase in the levels TNF-α, IL-6, and NK-κB, as shown in **Figure 4**. However, NGN demonstrated a significant reduction in the levels of pro-inflammatory cytokines compared to DASA treatment alone. The results suggest that NGN has the ability to alleviate the inflammatory response triggered by DASA, resulting in a decrease in the levels of pro-inflammatory cytokines.

### Effects of NGN on the Expression Levels of Apoptosis Markers

**Figure 5:**
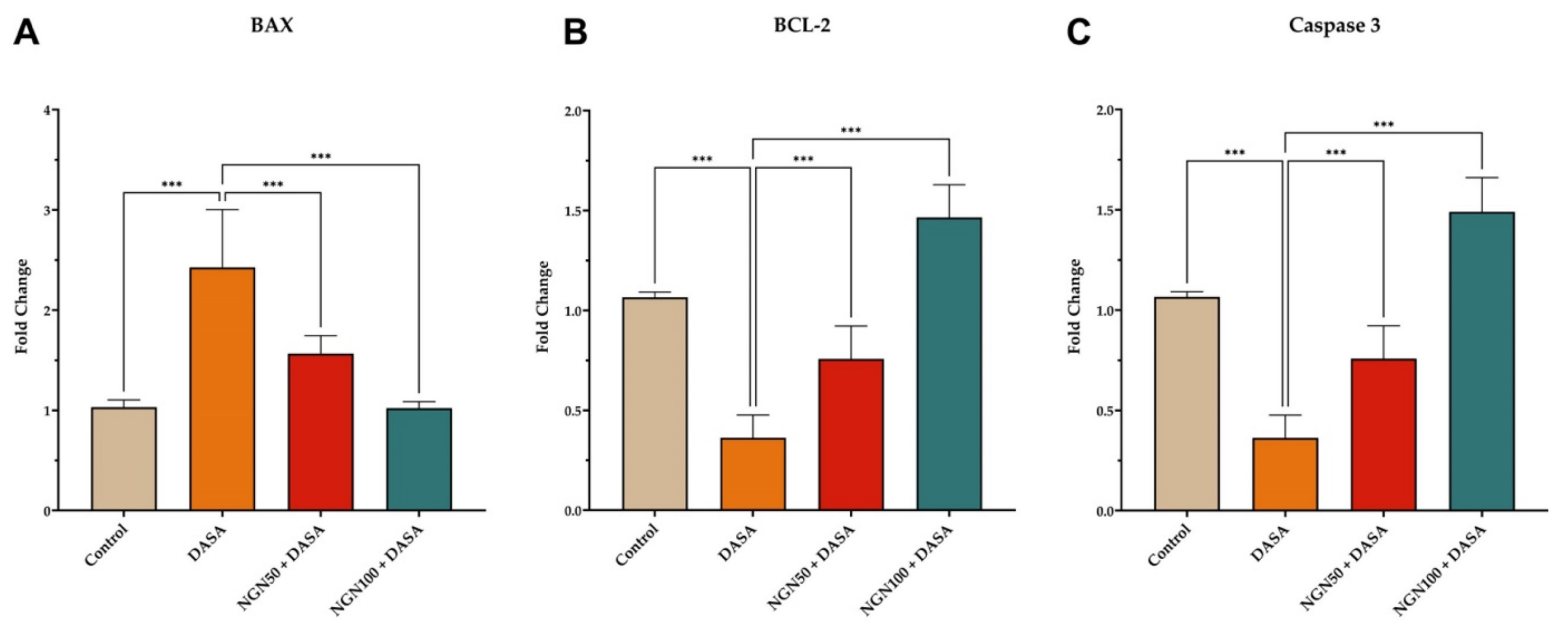
** Effects of NGN on the expression of apoptosis markers.** The figure depicts the expression levels of **(A)** BAX, **(B)** BCL-2, and **(C)** Caspase 3 in kidney tissue of mice under different treatment conditions. DASA treatment increases the expression of BAX, a pro-apoptotic protein, while decreasing the expression of anti-apoptotic proteins Caspase 3 and BCL-2, in kidney tissue. However, pretreatment with NGN at doses of 50 mg/kg and 100 mg/kg in combination with DASA reverses these effects. It leads to an increase in BCL-2 and Caspase 3 expression, while reducing BAX expression in the kidney tissue. This indicates that NGN may have protective effects by modulating the expression of apoptotic proteins, potentially counteracting the pro-apoptotic effects of DASA.

The impact of NGN ± DASA treatment on the expression levels of BAX, BCL-2, and Caspase 3 in kidney tissue was also investigated. The results revealed that DASA treatment led to an increase in BAX expression, a pro-apoptotic protein, while reducing the expression of Caspase 3, an executioner Caspase, and BCL-2, an anti-apoptotic protein, in kidney tissue **(Figure 5)**. These findings suggest that DASA may promote apoptosis in the kidneys. Conversely, pretreatment with NGN (50 and 100 mg/kg) in combination with DASA resulted in an increase in BCL-2 and Caspase 3 expression, while reducing BAX expression in the kidney tissue **(Figure 5)**. This indicates that NGN may exert protective effects by modulating the expression of apoptotic proteins, potentially counteracting the pro-apoptotic effects of DASA.

## Discussion

Drug-induced nephrotoxicity is a common complication associated with tyrosine kinase inhibitors 19. The mechanisms underlying such toxicity are diverse, involving direct renal injury, immune-mediated damage, inflammation, and changes in kidney hemodynamics 20. In this study, we investigated the nephroprotective effects of naringenin (NGN) pretreatment in mice subjected to dasatinib (DASA) treatment.

The results of this study demonstrated that DASA significantly elevated serum levels of creatinine, BUN, lactate dehydrogenase, and uric acid, indicating the development of nephrotoxicity. These findings are consistent with previous studies that have shown the nephrotoxic effects of DASA 19,21. In contrast, pretreatment with NGN resulted in a significant reduction in the serum levels of these markers. The extend of reduction was found to be inversely proportional to the doses of NGN, suggesting a dose-dependent protective effect of NGN against DASA-induced renal damage. These findings align with prior studies that have demonstrated the protective effects of NGN against different forms of renal damage 22,23

Inflammation plays a crucial role in the progression of kidney damage. Our results show that DASA treatment led to the upregulation of pro-inflammatory cytokines, such as NF-κB, TNF-α, and IL-6 suggesting the development of kidney inflammation. However, pretreatment with NGN showed a significant reduction in the expression of these pro-inflammatory cytokines, indicating the potential of NGN to attenuate kidney inflammation and subsequent damage. NGN plays a significant role in reducing inflammation by modulating the NF-κB signaling pathway. By inhibiting the activation of NF-κB, NGN can decrease the expression of pro-inflammatory cytokines, including TNF-α 24. This reduction in TNF-α levels is crucial, as TNF-α contributes to kidney damage through multiple mechanisms 25. First, it induces apoptosis or programmed cell death in renal cells, resulting in functional tissue loss. Second, TNF-α stimulates the production of other inflammatory mediators and chemokines, exacerbating the inflammatory response in the kidney and leading to recruitment of inflammatory cells and further tissue damage. Additionally, TNF-α increases the permeability of the glomerular filtration barrier, causing proteinuria, a common sign of kidney disease. TNF-α also contributes to renal fibrosis, characterized by excessive accumulation of extracellular matrix proteins, which leads to scarring and loss of kidney function.

Both TNF-α and IL-6 are critical mediators of the inflammatory response, and their elevated levels are believed to be due to the overexpression of cyclooxygenase-2 26. Several studies have shown that NGN has anti-inflammatory properties and can reduce the expression of pro-inflammatory cytokines, including TNF-α and IL-6 27,28. For example, a study showed that a blueberry-enriched diet improved renal function and reduced oxidative stress in metabolic syndrome animals by modulating the TLR4-MAPK signaling pathway, which is involved in the production of pro-inflammatory cytokines 29. Additionally, targeting endogenous kidney regeneration using anti-IL-11 therapy has been demonstrated to reduce inflammatory gene expression and improve renal function in both acute and chronic kidney disease models 30. Furthermore, fisetin, a flavonoid with anti-inflammatory properties, was found to ameliorate renal fibrosis by inhibiting SMAD3 phosphorylation, oxidative damage, and inflammation in ureteral obstructed kidney in mice 31

Apoptosis, a tightly regulated programmed cell death process, plays a crucial role in renal injury 32. Our results demonstrated that DASA treatment led to a significant increase in the expression of the pro-apoptotic protein BAX, while concomitantly reducing the expression of Caspase 3, an executioner caspase, and BCL-2, an anti-apoptotic protein, in kidney tissue. The upregulation of BAX in response to DASA treatment provides evidence for the activation of apoptotic pathways in renal cells^33^. This activation may contribute to the progression of renal injury observed in DASA-treated subjects. Furthermore, the downregulation of Caspase 3 and BCL-2 further supports the involvement of apoptosis in DASA-induced renal toxicity 33. In contrast, the pretreatment with NGN appears to counteract the pro-apoptotic effects of DASA. The increased expression of BCL-2 suggests that NGN may inhibit apoptosis by promoting cell survival mechanisms, while the upregulation of Caspase 3 indicates a potential role of NGN in regulating apoptosis in the kidneys. Several studies have shown that NGN has anti-apoptotic properties and can reduce the expression of BAX, while increasing the expression of BCL-2 34. For example, a study found that NGN pretreatment significantly reduced the expression of BAX and increased the expression of BCL-2 in a rat model of renal ischemia-reperfusion injury 35. Another study showed that NGN treatment reduced the expression of pro-apoptotic proteins and increased the expression of anti-apoptotic proteins in a rat model of diabetic nephropathy 36.

Several studies have shown that oxidative stress is involved in the pathogenesis of numerous chronic-degenerative disorders, including renal diseases 37-39. Oxidative stress is a phenomenon caused by an imbalance between production and accumulation of oxygen reactive species (ROS) in cells and tissues. This imbalance leads to the recruitment of inflammatory cells and the production of pro-inflammatory cytokines, initiating an inflammatory response that can progress to fibrosis and ultimately result in the loss of organ function 40. In this study, the administration of DASA resulted in increased oxidative stress, as indicated by elevated MDA levels and reduced CAT and GSH levels in the serum. However, pretreatment with NGN significantly reduced MDA levels and increased CAT and GSH levels compared to DASA treatment alone, indicating the antioxidant properties of NGN and its ability to mitigate DASA-induced oxidative stress.

These findings suggest that NGN has the potential to protect against DASA-induced nephrotoxicity by restoring the balance between ROS production and antioxidant defense mechanisms. It is plausible that NGN may engage the Nrf2/ARE (Nuclear factor erythroid 2-related factor 2/Antioxidant Response Element) signaling pathway, which plays a critical role in cellular defense against oxidative stress 41. Activation of Nrf2 is known to upregulate the expression of several antioxidant enzymes, including glutathione peroxidase and superoxide dismutase, thereby mitigating oxidative damage in renal tissues. By activating the Nrf2 pathway, NGN may enhance the cellular antioxidant capacity, leading to a reduction of oxidative stress^41^,^42^. This mechanism could explain the observed reduction in oxidative markers and the restoration of antioxidant enzyme levels in the presence of NGN. Furthermore, the activation of Nrf2 may also have additional protective effects, such as reducing inflammation and promoting cellular repair processes, which are crucial in the context of DASA-induced nephrotoxicity.

Despite the promising findings of our study, several limitations should be acknowledged. First, the relatively small sample size of experimental animals was primarily due to regulations set by Institutional Animal Care and Use Committee (IACUC), which governing the conduct of toxicity studies. These regulations are designed to ensure ethical treatment and minimize the number of animals used in such studies. This smaller sample size can lead to reduced statistical power, making it more challenging to detect significant effects or differences, and can limit the ability to generalize results to broader populations 43. However, it is noteworthy that even with this limitation, the protective effect of NGN against DASA-induced nephrotoxicity was still evident. This suggests that NGN may have a significant impact on renal function, warranting further investigation. Second, our study employed a single dose of DASA, which does not reflect the clinical practice where patients typically receive repeated doses of DASA 44. While focusing on a single dose allowed us to primarily address acute nephrotoxicity, it does not capture the distinct mechanisms of renal impairment associated with chronic DASA exposure that can lead to cumulative damage and prolonged inflammatory responses. Additionally, we did not investigate the potential impact of DASA dosing frequency on the nephroprotective effects of NGN. Given that patients treated with DASA 100 mg once daily experienced fewer adverse compared to those on a 70 mg twice-daily regimen, it raises the question of whether a more stable dosing schedule could enhance the protective effects of NGN against nephrotoxicity^45^. The pharmacokinetics and pharmacodynamics of DASA may differ significantly with chronic administration, potentially resulting in cumulative nephrotoxic effects as demonstrated in a case study of a 52-year-old woman with chronic myeloid leukemia, where prolonged DASA treatment caused progressive proteinuria and renal impairment 46. This continuous exposure may exacerbate renal injury and alter the pharmacokinetics of both DASA and NGN, highlighting the need for further exploration of their interactions over time. Third, our study lacked a detailed mechanistic investigation into the pathways involved in NGN nephroprotective action. Therefore, future research should consider using specific pathway inhibitors or knockout models targeting inflammation and apoptosis pathways to conduct more comprehensive studies that elucidate the underlying mechanisms of NGN's effects. Additionally, future studies should incorporate multiple dosing regimens to better simulate clinical conditions and assess the long-term effects of NGN on renal function.

## Conclusion

Our study demonstrates the potential of NGN as a nephroprotective agent in mitigating DASA-induced renal damage. NGN effectively reduced the serum levels of nephrotoxicity markers, attenuated inflammation, modulated apoptotic protein expression, and mitigated oxidative stress. These findings provide valuable insights into the molecular mechanisms underlying DASA-induced nephrotoxicity and highlight the therapeutic potential of NGN in protecting against renal damage. Further investigations are warranted to elucidate the precise mechanisms of action and evaluate the translational potential of NGN in clinical settings.

## Figures and Tables

**Table 1:** Scoring System for Histopathological Assessment of Kidney Damage.

Score	Field Percentage	Description
0	0%	No abnormalities observed
1	<20	Minimal abnormalities
2	20% - <35%	Mild abnormalities
3	35% - <50%	Moderate abnormalities
4	50% - < 75%	Severe abnormalities
5	75% - <90%	Very severe abnormalities
6	90 % - 100 %	Extreme abnormalizes

**Table 2:** ** Semiquantitative analysis of histopathological changes of kidney tissues.** Histopathological scores, ranging from 0 (indicating no damage) to 4 (indicating ≥50% involvement), are presented as median values with interquartile ranges (IQR) for each experimental group. Significant differences among the groups were evaluated using the Kruskal-Wallis test.

Histopathological Characteristic	Group				P value
	Control	DASA	NGN 50 + DASA	NGN 100 + DASA	
Neutrophil Infiltration	0 (0-0.5)	2 (1-3.5)	1 (0-2.5)	0 (0-0.5)	Ns
Eosinophilic Infiltration	0 (0-0.5)	2 (2-4)	0 (0-2.5)	1 (0-1)	P<0.05
Lymphocyte and Plasma Cell Infiltration	0 (0-1)	4 (2.5-5.5)	2 (1-3)	2 (0-2.5)	P<0.01
Mesangial Hypercellularity	0 (0-0.5)	4 (2-5)	2 (0-3)	1 (0-1.5)	P<0.05
Glomerular Space Enlargement	0 (0-0)	5 (4-6)	2 (0.5-2.5)	1 (0.5-2)	P<0.01
Dilated Tubules	0 (0-0)	4 (3-5)	3 (1-3.5)	2 (1-2.5)	P<0.01
Score	0 (0-0.25	4 (2.5-4.25)	2 (1.5-2.5)	1 (0.5-1.5)	P<0.001
